# Beyond mean-field age-structured models: degree heterogeneity and cross-country variability in epidemic risk

**DOI:** 10.1098/rsif.2026.0225

**Published:** 2026-10-21

**Authors:** Yinuo Qian, Chengyi Xia, Kazuki Kuga

**Affiliations:** ^1^ School of Control Science and Engineering, Tiangong University, Tianjin 300387, People's Republic of China; ^2^ School of Artificial Intelligence, Tiangong University, Tianjin 300387, People's Republic of China; ^3^ Interdisciplinary Graduate School of Engineering Sciences, Kyushu University, Kasuga-koen, Kasuga-shi, Fukuoka 816-8580, Japan; ^3^ Faculty of Engineering Sciences, Kyushu University, Kasuga-koen, Kasuga-shi, Fukuoka 816-8580, Japan

**Keywords:** edge-based compartmental modelling, age-structured compartment model, epidemic risk

## Abstract

Age structure is a fundamental determinant of infectious disease transmission, but most applications of age-specific contact matrices remain within mean-field compartmental frameworks based on population-averaged mixing. Such models describe average contact rates between age groups but do not explicitly represent variability in individual contact numbers or the network topology generated by age-dependent interactions. In this study, we combined age-stratified contact matrices with stochastic network-based epidemic simulations to investigate how demographic composition and contact structure shape epidemic dynamics across countries. Using the contact matrices developed by Prem *et al.* (Prem *et al.* 2017 *PLoS Comput. Biol.* **13**, e1005697 (doi:10.1371/journal.pcbi.1005697)), we constructed age-structured contact networks under a unified transmission rate, enabling cross-country comparisons that isolate structural effects from pathogen-specific differences. Stochastic susceptible–infected–recovered simulations on the generated networks revealed marked cross-country variation in final epidemic size and age-specific infection burden, even under identical epidemiological parameters. Countries with youthful populations and strong cross-age connectivity showed larger epidemic sizes, whereas countries with more stratified or ageing contact structures exhibited more constrained transmission. We further used an edge-based compartmental model as an analytical approximation to interpret the simulation results, derive *R*_0_ and assess the consistency between network simulations and theoretical expectations. The results show that *R*_0_ alone does not uniquely determine final epidemic size, because degree heterogeneity and age-specific network connectivity strongly influence epidemic outcomes. Analyses of school- and workplace-contact removal further demonstrated that intervention effectiveness depends on country-specific demographic and contact structures. This framework provides a data-driven approach for evaluating structure-dependent epidemic risks and highlights the importance of incorporating age-dependent network topology into epidemic modelling and intervention assessment.

## 1. Introduction

The spread of infectious diseases is influenced not only by the biological characteristics of pathogens but also by the social structures within which hosts are embedded [1]. Large-scale epidemiological studies have demonstrated that the transmission patterns of respiratory infections, human immunodeficiency virus (HIV) and other infectious diseases vary substantially across countries and regions [2]. Such variation cannot be fully explained by disease-specific factors alone and is closely associated with structural factors, such as population composition and patterns of daily social contact [3]. Traditional epidemiological modelling approaches, such as compartmental susceptible–infected–recovered (SIR) and susceptible–exposed–infectious–recovered model models based on the homogeneous-mixing assumption, provide a foundational framework for understanding transmission dynamics [4]. However, by assuming that all individuals interact uniformly, these models neglect the pronounced heterogeneity in human contact behaviours observed in real populations. Capturing this heterogeneity remains a central challenge in epidemic modelling [5,6], as infectious disease transmission is inherently a social process; pathogens rely on human interactions to propagate [7,8]. Consequently, the structure of these interactions—who meets whom, how frequently and in what context—affects the spread of diseases [9]. Age plays a uniquely important role among the various sources of heterogeneity. Age influences not only physiological susceptibility and immunity but also contact behaviour, which is shaped by school, work, family and social life [10–12]. This makes age-structured modelling a corner-stone of modern epidemiology, particularly in the context of airborne and droplet-transmitted diseases [13].

With the development of complex network science, social contact patterns have been increasingly modelled by treating individuals as nodes and their interactions as edges, thus providing a more realistic representation of disease transmission than homogeneous-mixing assumptions. The study of interaction behaviours within populations has become a central topic in interdisciplinary research spanning epidemiology, network science and spread dynamics [14,15]. A wide range of studies has demonstrated that network topology, community structure, temporal variation and adaptive behaviour can substantially alter epidemic outcomes, including outbreak size, epidemic thresholds and the emergence of superspreading events [16–27]. Despite these advances, many existing network-based epidemic models rely on synthetic or stylized network constructions, which are often based on assumed topological features or simplified mixing rules. Although such approaches are valuable for theoretical exploration, they may fail to capture the heterogeneity of real-world population contact behaviour, particularly when contact patterns are shaped by demographic and social factors.

Previous studies have similarly sought to integrate empirical contact or mobility data into epidemic models, demonstrating the importance of realistic interaction structures for understanding disease spread [28–31]. Along the critical dimension of age structure, substantial progress has been made through age-structured compartmental and network-based models, which have consistently shown that population age composition plays a key role in shaping transmission dynamics and disease burden [32–34]. Nevertheless, constructing epidemic models that simultaneously capture individual heterogeneity, age structure and realistic contact patterns remains challenging.

Over the past two decades, large-scale empirical studies have accumulated detailed data on age-specific contact patterns across diverse countries and cultural settings. One of the most comprehensive efforts is the work of Prem *et al.*, who extended the POLYMOD study [35] to generate age-stratified contact matrices for 152 countries [36]. These age-specific contact matrices have served as a foundational data source for a wide range of epidemiological studies [37–41].

Building on this framework, previous research used Prem's contact matrices to investigate how age structure shapes epidemic dynamics. For example, Vizi *et al.* [42] developed an age-group sensitivity analysis framework to quantify the impact of contact patterns across different age groups on key epidemic model outputs and identify the age groups most sensitive to disease transmission, thereby providing a basis for targeted interventions. Matrajt *et al.* [43] incorporated Prem's age-specific contact matrix as a core heterogeneous-mixing parameter in an age-structured transmission model to quantify the force of infection among age groups, thereby capturing realistic social contact structures at a population-averaged level and providing a practical basis for optimizing vaccine allocation strategies. Similarly, Moore *et al.* [44] incorporated the age-specific social contact matrix, M, proposed by Prem *et al.* as a core heterogeneous-mixing parameter in their multi-country age-structured compartmental model, enabling quantification of contact frequencies and infection forces between different age groups within each country.

By construction, the contact matrices proposed by Prem *et al.* [36] represent the average number of daily contacts between individuals in each age-group pair. This representation has enabled age-structured compartmental models to estimate key epidemiological quantities, such as the basic reproduction number and expected attack rates. Consequently, most applications of these contact matrices have remained within the mean-field compartmental modelling paradigm, in which contact patterns are aggregated into average mixing rates at the group level. Although this framework has been widely adopted, it does not explicitly represent individual-level contact heterogeneity within each age group or the underlying structural and topological properties of age-structured contact networks, such as variability in individual contact numbers and the resulting network connectivity patterns. Epidemic models on networks provide a natural framework for incorporating such heterogeneity and for linking microscopic contact structure to epidemic outcomes [45].

This individual-level variation in contact behaviour is not merely a statistical detail. Both empirical evidence and network theory have demonstrated that epidemic dynamics are strongly shaped by the distribution of contact degrees rather than by their average alone. Individuals with high contact degrees are more likely to become infected and transmit the disease to others, leading to super-spreading events and increasing variability in outbreak sizes [46,47]. Because epidemic outcomes are so sensitive to how contacts are distributed, intervention strategies, such as targeted vaccination, priority testing and age-specific control measures, depend not only on the average number of contacts individuals have [48,49] but also on how those contacts are distributed across the population. In settings where individual contact behaviours vary widely owing to occupation, socio-economic status or urban density, relying solely on group-level average contact rates may, therefore, mischaracterize epidemic risk and burden.

To address this limitation, we constructed age-structured contact networks from the empirical contact matrices provided by Prem *et al.* and performed stochastic SIR simulations on these networks. These simulations provide the main numerical evidence for country- and age-specific epidemic outcomes in the generated contact networks. In parallel, we used an edge-based compartmental model (EBCM), following the framework of Miller *et al.* [50], as an analytical approximation for age-structured networks with degree heterogeneity. The EBCM-based quantities, including *R*_0_ and final-size predictions, are used to interpret the simulation results and to assess the consistency between stochastic network dynamics and theoretical expectations. Using this combined framework, we evaluated cross-country differences in epidemic size, age-specific infection burden and the effects of setting-specific contact removal. Our results highlight that epidemic outcomes cannot be fully explained by population-averaged contact rates alone, and that age-dependent network structure and degree heterogeneity should be explicitly considered when assessing epidemic risk and intervention effects.

## 2. Material and methods

### 2.1. Age-specific contact matrices and symmetrization

We use age-specific contact matrices estimated by Prem *et al.* [36], which provide the average daily number of contacts between individuals in different age groups in 152 countries. In this dataset, the population of each country is divided into 16 age groups, each with a 5-year interval (0–4, 5–9, …, 75–79 years).

Let *C_ij_* denote the average number of daily contacts an individual in age group *i* has with individuals in age group *j*. In general, the empirical contact matrix is asymmetric, that is $C_{ij}\neq C_{ji}$, because it reflects reported contacts rather than realized mutual interactions. However, when constructing undirected contact networks or applying degree-based analytical models, a reciprocity condition is required to ensure the consistency of total contacts between age groups [48].

To enforce this condition, we adopt a standard symmetrization procedure and define the symmetrized contact matrix $\tilde{C}$ as

(2.1) $${\tilde{C}}_{ij}=\frac{N_{i}C_{ij}+N_{j}C_{ji}}{2N_{i}},$$

where *N_i_* and *N_j_* denote the population sizes of the age groups *i* and *j*, respectively. This symmetrization ensures that the total number of contacts between the age groups satisfies the reciprocity condition.

(2.2) $$N_{i}\;{\tilde{C}}_{ij}=N_{j}\;{\tilde{C}}_{ji}.$$

Thus, matrix $\tilde{C}$ represents the population-consistent average number of contacts between age groups and serves as the target mixing structure for both network construction and analytical modelling throughout this study.

A note on temporal consistency between demographic and contact data is warranted. The age-specific contact matrices from Prem *et al.* [36] ultimately derive from the POLYMOD survey conducted during 2005–2006 [35], whereas the population age distributions used in our network construction correspond to the year 2023. Our aim is not to reconstruct epidemic dynamics of the past decade but to compare, under a unified framework, how current demographic structures (2023) interact with baseline age-specific mixing patterns to shape epidemic outcomes across countries. Evidence suggests broad temporal persistence in age-specific social mixing patterns: the BBC Pandemic study [49] compared UK contact data from 2017 to 2018 with the original POLYMOD data and found similar age-specific patterns for most age groups. Therefore, we regard this combination as a reasonable approximation for assessing contemporary demographic effects under a common contact-pattern baseline. Nevertheless, we acknowledge this temporal mismatch as a limitation, and future updates of contact matrices will improve accuracy when more recent country-specific surveys become available.

### 2.2. Construction of the age-structured network model

Using the symmetrized contact matrix $\tilde{C}$ and country-specific age distributions, we constructed age-structured contact networks that reproduce the prescribed average contact patterns at the group level, while allowing for individual-level degree heterogeneity. The construction can be interpreted as a type-structured random graph, closely related to multi-type random graph and configuration-model approaches used in network epidemiology [51–53].

The network construction proceeds as follows:

**Step 1: Node initialization.**
  We initialize a network consisting of *N* nodes without edges, where each node represents an individual.
**Step 2: Age-group assignment.**
  Nodes are assigned to age groups based on the empirical age distribution for each country (year 2023) [54]. Consequently, the number of nodes in age group *i* equals *N_i_*, satisfying $\sum_{i}N_{i}=\;N$.
**Step 3: Within-group edge generation.**
  For each age group *i*, edges among nodes within the group are generated independently following a Poisson degree distribution with mean degree $\langle k\rangle \;={\tilde{C}}_{ii}\;$. This corresponds to constructing an Erdős–Rényi subnetwork within each age group that reproduces the prescribed average number of intragroup contacts.
**Step 4: Between-group edge generation.**
  For each pair of distinct age groups i≠j, edges between nodes in group *i* and nodes in group *j* are generated independently with probability $p_{ij}={\tilde{C}}_{ij}/N_{j}$.
  This choice ensures that the expected number of contacts from a node in group *i* to nodes in group *j* equals ${\tilde{C}}_{ij}$, and that the total number of intergroup edges satisfies the reciprocity condition imposed by the symmetrized contact matrix.
  Through this procedure, the resulting network reproduces the empirical age‑specific mixing structure at the level of mean contact while allowing stochastic variability in individual contact numbers, arising from the random edge-generation processes inherent to the configuration‑model‑based construction (Steps 3 and 4). A schematic of the network construction process is shown in figure 1.

**Figure 1.**
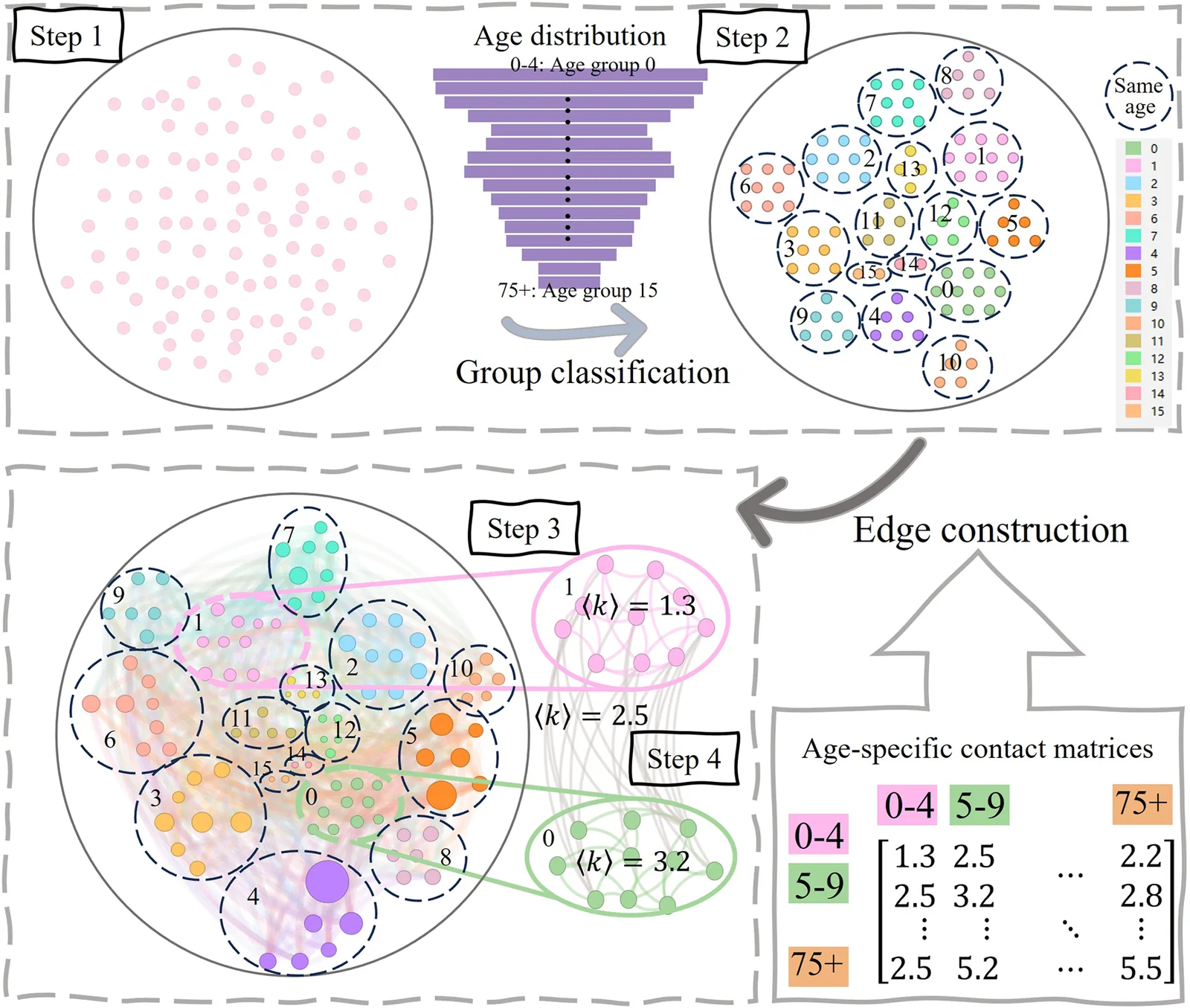
Schematic illustration of the network construction process based on the age contact matrix and the 2023 age distribution data of selected countries. The network is constructed in four steps. Step 1: Node initialization – start with *N* isolated nodes (individuals). Step 2: Age-group assignment – assign each node to an age group *i* according to the country's 2023 age distribution, with group size *N_i_* satisfying $\sum_{i}N_{i}=\;N$. Step 3: Within-group edge generation – for each age group *i*, generate edges among its nodes independently following a Poisson degree distribution with degree $\langle k\rangle \;{\tilde{C}}_{ii}$, producing an Erdős–Rényi subnetwork that reproduces the prescribed average intragroup contact number. Step 4: Between-group edge generation – for each pair of distinct groups i≠j, add an edge between a node in group *i* and a node in group *j* independently with probability $p_{ij}={\tilde{C}}_{ij}\;/N_{j}$. In the schematic, individuals enclosed by each dashed circle belong to the same age group, and the numbers indicate the age group labels.

### 2.3. Disease transmission model and stochastic simulation

Disease transmission in the constructed networks was modelled using the classical SIR framework. Each node represents an individual and can be in one of three epidemiological states: susceptible (S), infected (I) or recovered (R). Transmission occurs through the network edges. When a susceptible individual is connected to an infected neighbour, infection occurs at a rate *β*. Infected individuals recover at a rate *γ* and transition to the recovered state. To accurately capture stochastic effects and asynchronous event timing, we simulate the SIR dynamics using the Gillespie algorithm [45]. At each simulation step, infection and recovery events are selected probabilistically according to their rates, and the system time is advanced accordingly. This approach ensures consistency with the continuous-time formulation assumed in the analytical model. All stochastic simulations were run for 30 independent realizations per country and scenario. The final epidemic size was recorded for each realization, and the reported values are the means over all realizations. We record the time evolution of the number of susceptible, infected and recovered individuals in each age group, as well as the final epidemic size at both the population and age-group levels.

## 3. Theoretical model and numerical set-up

### 3.1. Edge-based compartmental model for age-structured contact networks

In the standard age-structured SIR model [45–55], transmission depends on the average number of contacts between the age groups. However, this ignores the heterogeneity in the number of contacts within each age group. To address this, we adopted the framework proposed by Miller *et al.* [50], which incorporates assortative mixing by type within contact-degree distributions.

Let *P_ij_*(*u*) denote the probability that an individual in age group *i* has exactly *u* contacts with individuals in age group *j*. We assume that contacts with different age groups are independent. For an individual in group *i*, denoted by *k_j_*, the number of contacts this individual has with age group *j*, where *k_j_* follows the distribution *P_ij_*. The individual's total degree is $k\;=\sum_{j=1}^{n}k_{j}\;$, where *n* is the number of age groups, that is *n* = 16, corresponding to the 16 five-year age groups. The probability that such an individual has total degree *k* is given by the convolution in equation (3.1).

(3.1) $$P_{i}\;\left(k\right)=\sum_{\left\{k_{j}\right\}:\sum_{j=1}^{n}k_{j}=k}\prod_{j=1}^{n}P_{ij}\left(k_{j}\right).$$

That is, the sum runs over all non-negative integer tuples ($k_{1},\ldots ,k_{n}$), the components of which sum to the total degree *k*. In practice, each *P_ij_*(*u*) is fitted using a binomial or negative binomial distribution with mean $E[k_{ij}]\;={\tilde{C}}_{ij}\;$, as informed by the empirical contact matrices (e.g. Prem *et al.*). The corresponding probability generating functions (PGFs) are shown in equation (3.2).

(3.2) $$\psi_{ij}\;\left(x\right)=\sum_{k=0}^{\infty }P_{ij}\;\left(k\right)\;x^{k},\;\psi_{i}\;\left(x\right)=\prod_{j=1}^{n}\psi_{ij}\;\left(x_{j}\right),$$

where $x\;=\;(x_{1},\ldots ,x_{n})$ encodes contacts to all age groups.

This construction defines a multi-type configuration network, where each node is assigned a degree vector $\left(k_{i1},\ldots ,k_{in}\right)$, and the edges are matched consistently with the mixing proportions implied by ${\tilde{C}}_{ij}$.

The PGFs allow for the natural derivation of the basic reproduction number *R*_0_. The first derivative yielded the mean number of contacts, as shown in equation (3.3).

(3.3) $$\psi_{ij}^{{}^{\prime }}\;\left(1\right)=k_{ij}.$$

However, the second derivative encodes the excess degree and variance.

Let *T* = *β*/(*β* + *γ*) be the per-edge transmission probability. Element *K_ij_* of the next-generation matrix is defined as the expected number of secondary infections in group *i* caused by an infected individual in group *j* following an infection along a randomly chosen edge, as given in equation (3.4).

(3.4) $$K_{ij}=\;T\;\frac{\psi_{ij}^{{}^{\prime \prime }}\left(1\right)}{\psi_{ij}^{{}^{\prime }}\left(1\right)}.$$

The basic reproduction number is then given by the spectral radius of **K**: *R*_0_ = *ρ*(**K**), where *ρ*( · ) denotes the spectral radius—that is the largest absolute eigenvalue. Epidemiologically, *R*_0_ serves as a threshold parameter: if *R*_0_ > 1, the infection can invade and cause an epidemic; if *R*_0_ < 1, the infection cannot sustain itself and will die out. This formulation generalizes classical age-structured SIR models by incorporating heterogeneity in contact numbers and assortative mixing between age groups [45,56].

Let *θ*_*ij*_(*t*) denote the probability that an edge from a susceptible individual in group *i* to a neighbour in group *j* has not transmitted infection by time *t*. Under the locally tree-like assumption—a standard approximation for EBCMs—the infection states of neighbours can be treated as conditionally independent. Our generated networks are otherwise random and are expected to be approximately locally tree-like in the large-network limit, although finite-size effects and short cycles may exist. Therefore, we treat the EBCM as an approximation, the accuracy of which is evaluated by comparing its predictions with stochastic network-based simulations. Under this assumption, the fraction of susceptible individuals in group *i* is given by equation (3.5).

(3.5) $$S_{i}\left(t\right)\;=\psi_{i}\;\left(\theta_{i}\left(t\right)\right)\;=\prod_{j=1}^{n}\psi_{ij}\;\left(\theta_{ij}\left(t\right)\right).$$

For a randomly chosen edge from group *j* to a test node in group *i*, we define edge-level state probabilities $\phi_{S:ij},\;\phi_{I:ij},\;\phi_{R:ij}$, satisfying $\theta_{ij}=\phi_{S:ij}\;+\phi_{I:ij}+\phi_{R:ij}$.

The probability that a neighbour in group *j* is still susceptible and does not transmit the infection is given by equation (3.6).

(3.6) $$\phi_{S:ij}=\frac{\partial_{x_{i}}\psi_{j}\left(\theta_{j}\right)}{\partial_{x_{i}}\psi_{j}\left(1\right)}\;=\frac{\partial_{x_{i}}\psi_{ji}\left(\theta_{ji}\right)}{\partial_{x_{i}}\psi_{j}\left(1\right)}\;\prod_{m\neq i}\psi_{jm}\left(\theta_{jm}\right).$$

Recovery without transmission occurs with probability given by equation (3.7).

(3.7) $$\phi_{R:ij}=\frac{\gamma }{\beta }\;\left(1-\theta_{ij}\right).$$

Combining transmission and recovery yields the closed edge-level equation given by equation (3.8).

(3.8) $${\dot{\theta }}_{ij}=\;-\beta \;\theta_{ij}+\beta \frac{\partial_{x_{i}}\psi_{ji}\left(\theta_{ji}\right)}{\partial_{x_{i}}\psi_{j}\left(1\right)}\prod_{m\neq i}\psi_{jm}\left(\theta_{jm}\right)+\gamma \;\left(1-\theta_{ij}\right).$$

Together with equation (3.8), this system fully characterizes epidemic dynamics in age-structured configuration-model networks with heterogeneous and assortative contacts.

The EBCM framework also allows analytical derivation of the final epidemic size through the steady-state limit of the edge-level equations.

Let $\theta_{ij}^{*}=\underset{t\to \infty }{lim}\;\theta_{ij}(t)$ denote the probability that no transmission ever occurs along an edge from group *j* to group *i*. At epidemic equilibrium, ${\dot{\theta }}_{ij}=\;0.$

Rearranging gives the coupled fixed-point equations in equation (3.9).

(3.9) $$\theta_{ij}^{*}=\frac{\beta \frac{\partial_{x_{i}}\psi_{ji}\left(\theta_{ji}^{*}\right)}{\partial_{x_{i}}\psi_{j}\left(1\right)}\prod_{m\neq i}\psi_{jm}\left(\theta_{jm}^{*}\right)+\gamma }{\beta +\gamma }\;.$$

Once the fixed-point system is solved, the final susceptible fraction in age group *i* is obtained from equation (3.10).

(3.10) $$S_{i}^{\infty }=\psi_{i}\;\left(\theta_{i}^{*}\right)\;=\prod_{j=1}^{n}\psi_{ij}\;\left(\theta_{ij}^{*}\right),$$

where $\theta_{i}^{*}=\;(\theta_{i1}^{*},\ldots ,\theta_{in}^{*})$.

Assuming no initially recovered individuals, the final epidemic size in age group *i* is given by equation (3.11).

(3.11) $$R_{i}^{\infty }=\;1-S_{i}^{\infty }.$$

Let *w_i_* denote the demographic proportion of age group *i*, satisfying $\sum_{i\;=\;1}^{n}w_{i}=\;1.$

The overall final epidemic size in the population is, therefore, given by equation (3.12).

(3.12) $$R^{\infty }=\sum_{i=1}^{n}w_{i}\;R_{i}^{\infty }.$$

This result generalizes the classical mean-field final-size relation to heterogeneous age-structured configuration-model networks by explicitly incorporating degree heterogeneity and assortative mixing patterns through the multivariate generating functions.

### 3.2. Numerical set-up and parameter calibration

Prior to numerical simulations, we evaluated the theoretical steady states of the age-structured epidemic model for all countries using complete age-specific contact data from Prem *et al.* Countries for which reliable demographic or contact-matrix data could not be consistently obtained were excluded from the analysis.

In the subsequent simulations and theoretical calculations, we assumed that the per-contact transmission rate *β* is identical across countries, so that cross-country differences in epidemic outcomes arise solely from variations in age structure and contact patterns. To calibrate a common *β* value, we adopt the following procedure. First, a reference country is selected whose contact matrix exhibits a moderate spectral radius (neither extremely high nor extremely low). Second, a target final epidemic size for this reference country is specified, chosen within an intermediate range to avoid saturation of all countries at near-zero or near-full infection. Third, a numerical grid search over *β* is performed to find the value for which the theoretical final epidemic size predicted by the age-structured EBCM model for the reference country matches the chosen target. The resulting *β* is then used in all theoretical calculations and stochastic simulations for every country. All specific parameter values, the identity of the reference country, the target value, the grid search range and the data-processing steps are detailed in the ‘Results’ section.

## 4. Results

Before presenting the theoretical and simulation results, we specify the exact parameter values and data-processing choices used in this study. The transmission parameter *β* was calibrated using the EBCM framework, which is the main theoretical approximation used in this study. The calibrated value of *β* was not intended to represent a pathogen-specific empirical transmission rate but was introduced as a common transmission parameter to compare the effects of demographic structure and contact network topology across countries. The recovery rate is fixed at *γ* = 1/3 per day corresponding to a mean infectious period of 3 days. We set *γ* = 1/3 per day to define a representative epidemic time scale rather than to fit a specific pathogen. Because *β* is calibrated conditional on *γ*, the cross-country comparisons are governed primarily by the network structure and the resulting edge transmissibility, while the precise value of *γ* mainly affects epidemic timing.

For the calibration of *β*, India is selected as the reference country because its age-specific contact matrix has a near-median largest eigenvalue. A target final epidemic size of 0.8 is set for India. As a preliminary step, a broad range 0 ≤ *β* ≤ 0.4 was examined in EBCM-based sensitivity analyses to characterize the qualitative epidemic response of the model. This range spans the transition from subcritical or near-subcritical dynamics to a near-saturation regime. In the subcritical case, major outbreaks do not occur. In the near-saturation regime, most countries exhibit very large final epidemic sizes. Here, cross-country differences become difficult to distinguish. This preliminary exploration indicated that the calibrated transmission rate lies well below the saturation regime, which motivates and validates the narrower calibration range used below. Accordingly, the calibration of *β* is performed with a grid search over *β* ∈ [0.01, 0.1] (Step 0.001). This grid search yields *β* = 0.058. It is important to note that this calibrated transmission rate is obtained through a theoretical calibration procedure rather than empirical fitting to outbreak time series. No specific epidemic data were used to determine *β*. This approach ensures that our framework remains pathogen-agnostic and isolates the structural effects of age-specific contact patterns on transmission dynamics. This common transmission rate is then used for all countries in both theoretical predictions and network simulations. The original age-specific contact matrices contain 16 five-year age groups (0–4, 5–9, …, 75+).

For clearer identification of age-specific transmission patterns across countries and to facilitate the selection of representative countries, the 16 five-year age groups are first aggregated into four broader categories: younger (0–19 years), lower middle-aged (20–39 years), upper middle-aged (40–59 years) and older (60+ years). After identifying representative countries based on these aggregated categories, we then perform a more detailed analysis using the original 16 age groups to examine the fine-scale age-specific infection burden (figures 2, 7, 10 and 11).

**Figure 2.**
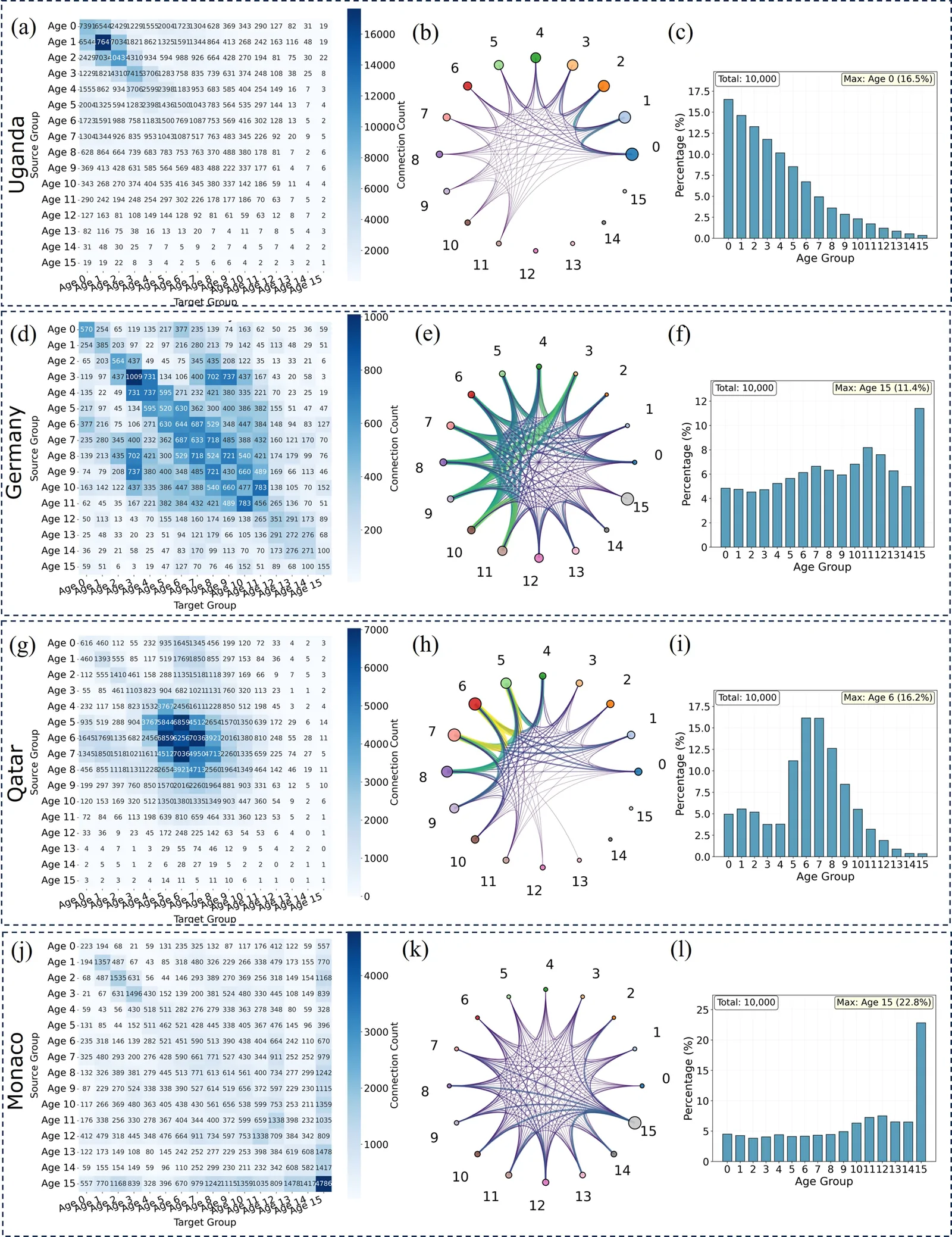
Structural comparison of age-structured contact networks in the four representative countries (Uganda, Germany, Qatar and Monaco). Each row corresponds to one country and presents network characteristics derived from its symmetrized age-specific contact matrix and demographic structure. The subfigures are organized as follows: Uganda (a–c), Germany (d–f), Qatar (g–i) and Monaco (j–l). For each country, the three columns show: (a), (d), (g) and (j) heat maps of age-specific contact intensities, where colour intensity represents the strength of within- and between-age-group connections; (b), (e), (h) and (k) chord diagrams illustrating intergroup connectivity, where chord thickness indicates the magnitude of connections between age groups, and node size on the outer ring is proportional to the population share of each age group; and (c), (f), (i) and (l) population age distributions, showing the relative proportion of each age group in the total population. Despite a unified construction framework, the resulting networks differ substantially across countries: Uganda and Qatar display high connectivity across all age groups, whereas Germany and Monaco show more compartmentalized structures, with notably lower contact intensity involving the oldest age group.

For each country, the aggregated final epidemic size for a category is computed as the population-weighted average of the constituent five-year age groups. For stochastic network simulations, the population size for each country is fixed at N=10000 nodes, with age-group sizes assigned according to each country's 2023 age distribution. For each country, 30 independent runs of the Gillespie algorithm are performed. Results are reported as the mean with its 95% confidence interval. Countries with incomplete or unreliable contact-matrix data are excluded from the analysis; the remaining 143 countries are used for the supplementary distribution plots, and four representative countries (Germany, Uganda, Qatar and Monaco) are selected for detailed presentation based on their distinct epidemic regimes.

### 4.1. Construction and analysis of age-structured networks in different countries

To elucidate the structural origins of the cross-country differences observed in the theoretical analysis, we construct explicit age-structured contact networks for four representative countries (Germany, Uganda, Qatar and Monaco). These networks were generated using symmetrized age-specific contact matrices and corresponding national age distributions for 2023.

To ensure computational tractability, while preserving the relative structural differences implied by the contact matrices, the population size for each country was fixed at 10 000 nodes. Because our objective is a comparative structural analysis rather than absolute population forecasting, this normalization allows for meaningful cross-country comparisons without altering the intrinsic mixing patterns encoded in the contact matrices.

Figure 2 shows the structural characteristics of the networks constructed using complementary representations, including heat maps of intergroup connectivity, chord diagrams of edge strengths and population age distributions. Although networks are generated within a unified construction framework, they exhibit pronounced structural differences across countries. Uganda displays dense intra- and inter-generational connectivity, particularly among younger age groups, which is consistent with the youthful demographic structure and high infection levels observed in the theoretical analysis. By contrast, Germany exhibited comparatively weaker cross-age connectivity and a more stratified mixing pattern, which structurally constrains large-scale transmission. Qatar exhibited strong connectivity concentrated in working-age groups, whereas Monaco maintained relatively substantial connectivity among older age groups. These structural differences provide a mechanistic explanation for the heterogeneous epidemic outcomes observed in mean-field and EBCM analyses.

To further quantify the global network structure, we analysed the degree distributions of the constructed networks. Although within- and between-group connections were generated using Poisson processes at the group level, heterogeneity in contact intensities across age groups led to substantial variability in individual connection probabilities.

Figure 3 compares the empirical degree distributions of the four country-specific networks with fitted Poisson and negative binomial models. Across all four countries, the empirical distributions are markedly right-skewed and overdispersed relative to the Poisson model. This is confirmed by the dispersion coefficients, which are all greater than 1 (Uganda: 3.59; Qatar: 3.33; Monaco: 2.08; Germany: 2.27), indicating variance substantially larger than the Poisson expectation. In addition, the negative binomial model consistently provides a better fit than the Poisson model, as shown by the smaller Kolmogorov–Smirnov (KS) [57] statistics in every country (Uganda: 0.0404 vs 0.1722; Qatar: 0.0184 vs 0.1505; Monaco: 0.0208 vs 0.1052; Germany: 0.0431 vs 0.1398). These results indicate that aggregation over heterogeneous age-specific contact rates naturally produces extra-Poisson variation in the global degree distribution, which is well captured by a negative binomial form.

**Figure 3.**
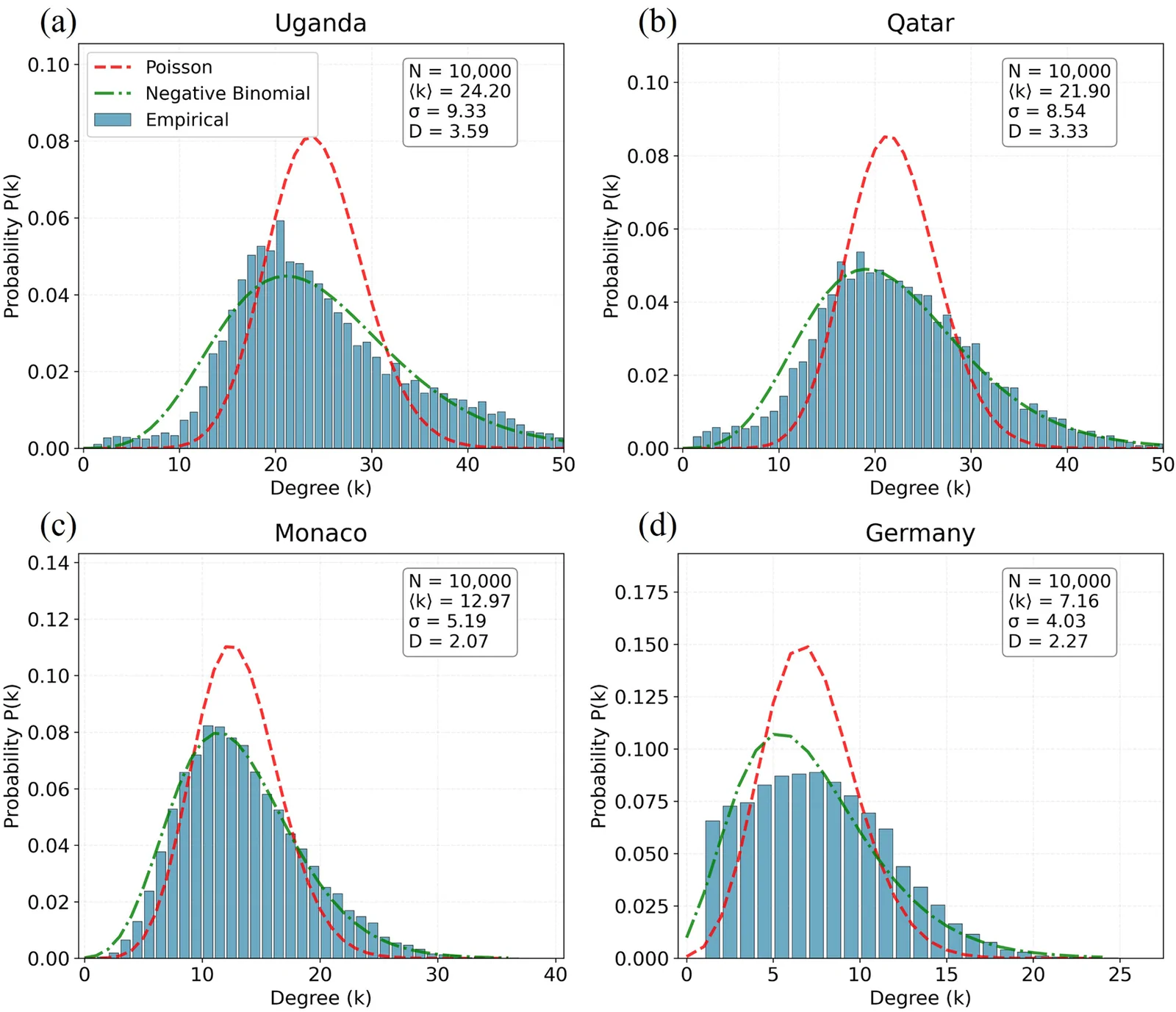
Degree distribution comparison of age-structured contact networks in the four representative countries. (a) is Uganda, (b) is Qatar, (c) is Monaco, and (d) is Germany. Empirical degree distributions obtained from the constructed networks (symbols or bars) are compared with fitted Poisson and negative binomial distributions (solid lines). σ is the standard deviation of the degree distribution, and *D* = σ^2^/*µ* is the dispersion coefficient. *D* > 1 indicates overdispersion (variance > mean), meaning the degree distribution deviates from a Poisson pattern owing to heterogeneous contact intensities across age groups. The aggregated degree distributions exhibit pronounced right skewness and overdispersion. The negative binomial distribution provides a substantially better fit than the Poisson distribution in all countries, reflecting the significant degree of heterogeneity inherent in the age-structured contact networks.

The magnitude of this heterogeneity varies across countries. Germany exhibited the lowest mean degree and a comparatively narrower degree distribution, consistent with its smaller epidemic potential in the simulated setting. Uganda and Qatar showed broader degree distributions and more extended upper tails, indicating a larger proportion of highly connected individuals who may facilitate sustained transmission. Monaco occupied an intermediate structural regime, while retaining relatively strong connectivity in older age groups, consistent with the age-specific infection patterns observed in the simulations.

To further assess the tail behaviour, figure 4 presents the same empirical degree distributions on a log–log scale. The distributions show clear downward curvature in the upper tail rather than an approximately linear scaling regime. The results are interpreted as heterogeneous, overdispersed count distributions arising from mixing across age groups with different effective contact rates. In this sense, the global network behaves as a mixture of Poisson-like processes, which naturally gives rise to a negative binomial pattern at the aggregate level.

**Figure 4.**
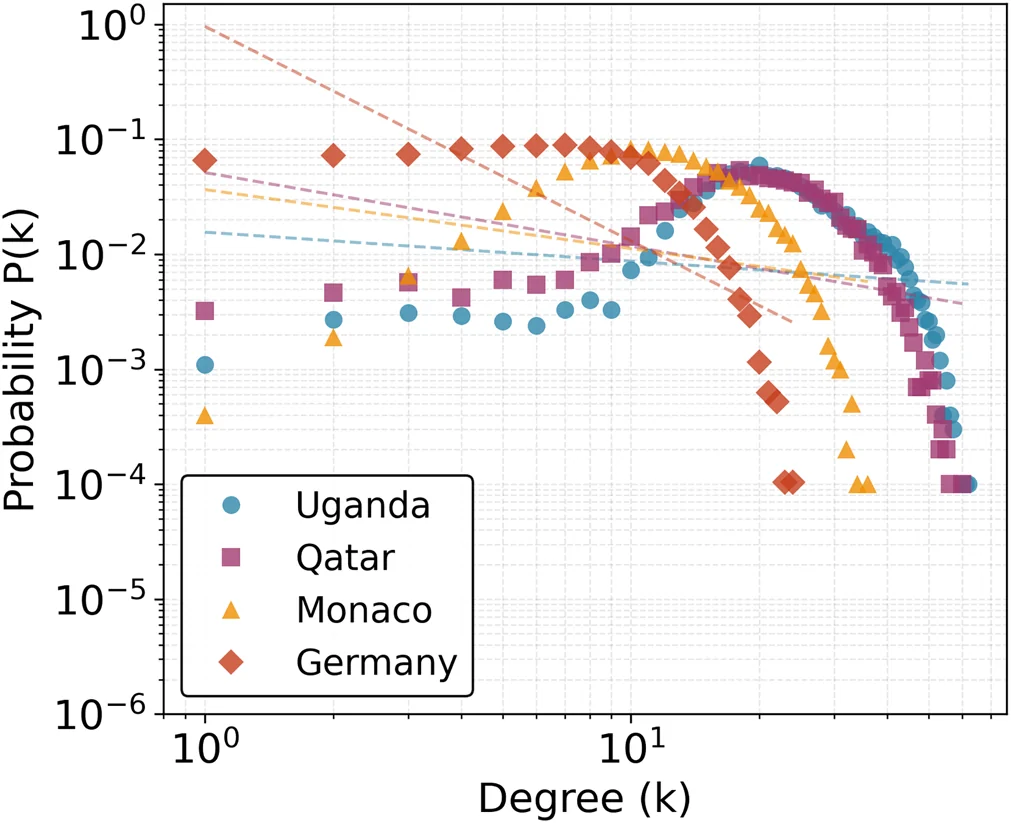
Log–log representation of the empirical degree distributions for the four representative countries. Each point denotes the observed probability of degree in the constructed age-structured contact network. The log–log plot shows that the upper tails are broader than those expected under a Poisson distribution but also display clear curvature rather than an approximately linear scaling regime. This indicates that the degree distributions are overdispersed and heterogeneous, yet not heavy-tailed in the strict sense. The pattern is consistent with aggregation over age groups with different contact intensities, producing a negative-binomial-like degree distribution at the population level.

These overdispersed count distributions can be connected to well-known continuous probability families studied in other modelling communities. The negative binomial distribution is closely connected to the Gamma distribution through its Poisson–Gamma mixture representation. Specifically, if latent individual contact intensities follow a Gamma distribution and contact counts are conditionally Poisson given those intensities, the resulting marginal count distribution is negative binomial. In the present network construction, however, we do not assume that individual or age-specific contact intensities are exactly Gamma-distributed. Rather, aggregation across age groups with different effective contact intensities produces heterogeneity in the underlying contact rates and can generate extra-Poisson variation in the aggregate degree distribution. Therefore, the Poisson–Gamma mixture provides a useful mechanistic interpretation of why a negative binomial distribution may approximate the resulting overdispersed degree distribution, rather than an exact description of our network-generation mechanism. Gamma-type contact distributions have been studied within several modelling communities, as have the mechanisms underlying their emergence [58–60]. Our empirical results can, therefore, be viewed as being consistent with, in an age-structured network setting, phenomena already identified theoretically in these related literatures. We further note that the KS statistic used here to compare empirical and fitted distributions coincides with the *L*^∞^ distance between their cumulative distribution functions. Although the KS statistic is not itself an optimal-transport metric, Wasserstein-type distances provide a complementary approach to comparing probability distributions. In one dimension, for example, the *W*_1_ distance corresponds to the integrated absolute difference between their cumulative distribution functions, whereas the KS statistic measures the maximum vertical separation. The contribution of this work lies not in the overdispersed form itself. Rather, it lies in constructing synthetic country-specific age-structured contact networks calibrated to empirical contact matrices. Using these networks, we quantify how the resulting heterogeneity shapes epidemic dynamics and intervention outcomes across countries and age groups.

These findings demonstrate that cross-country differences in epidemic size are not determined solely by the basic reproduction number *R*_0_ but are strongly shaped by the higher-order structural properties of the contact network. The degree of heterogeneity amplifies transmission by increasing the likelihood of highly connected individuals contributing disproportionately to the disease spread.

Thus, the structural analysis confirms that demographic composition and contact heterogeneity jointly determine the epidemic potential. Countries with youthful populations and dense cross-age mixing (e.g. Uganda) generate networks with higher connectivity and stronger overdispersion, promoting large epidemic sizes. Conversely, countries with ageing populations and more stratified mixing (e.g. Germany) produce structurally sparser networks that suppress widespread transmission. This structural perspective bridges the theoretical epidemic analysis and stochastic simulations, establishing a contact network topology as the fundamental driver of the observed cross-country heterogeneity.

### 4.2. Theoretical analysis of epidemic dynamics in representative countries

Using the calibrated transmission rate *β* = 0.058 and a fixed recovery rate *γ* = 1/3, together with the EBCM-based derivation of the final epidemic size described in §3.1 (equations 2.2–3.12), we evaluated the epidemic steady states for all countries with complete age-specific contact data provided by Prem *et al.* [36]. By applying identical transmission parameters across all countries, we isolated the structural effects of age composition and contact patterns independent of disease-specific transmission intensity. Therefore, differences in epidemic outcomes arise solely from variations in demographic structures and contact matrices.

Across all countries, substantial heterogeneity was observed in both the overall epidemic size and the age-specific distribution of infections. Germany consistently exhibited the smallest overall final epidemic size, whereas Uganda showed the largest, indicating marked cross-country differences in epidemic potential even under identical transmission parameters.

Using the four aggregated age categories defined above, the results revealed a pronounced contrast across countries. Among younger age groups (0–19 years), Uganda had the highest infection burden (0.976), whereas Germany had the lowest (0.349). In the lower-middle-aged group (20–39 years), Qatar had the highest infection rate (0.981), whereas Germany had the lowest (0.396). In the upper-middle-aged group (40–59 years), Uganda showed the highest infection rate (0.924), whereas Germany had the lowest (0.348). Among the older population (60+), Monaco exhibited the highest infection fraction (0.723), whereas Germany showed the lowest (0.097). These results demonstrate that age-specific infection burdens vary systematically across countries and are closely linked to underlying demographic composition and contact intensity.

Based on these findings, we selected four representative countries (Germany, Uganda, Qatar and Monaco) to illustrate the distinct epidemic regimes characterized by differences in the overall epidemic scale and age-specific infection burden. Figures 5 and 6 present the theoretical epidemic outcomes for these countries.

**Figure 5.**
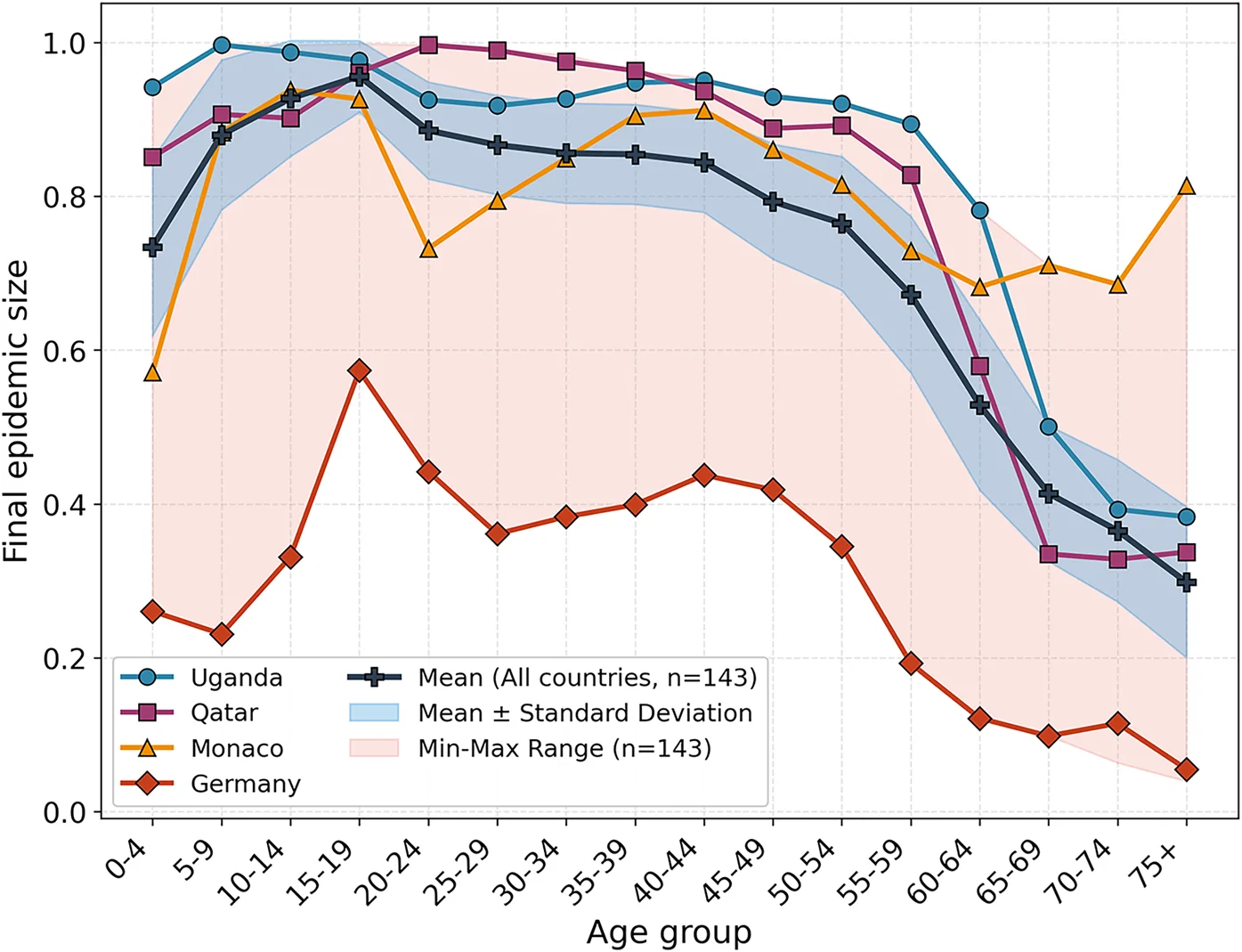
Age-specific final epidemic size across 16 age groups for four representative countries under a common transmission parameter (*β* = 0.058, *γ* = 1/3). Lines represent country-specific age-dependent final infection fractions, illustrating how demographic structure and contact patterns shape the distribution of epidemic burden across age groups. The black solid line with plus markers shows the mean across 143 countries (overall mean = 0.783, s.d. = 0.088) and red shading for the full min–max range (0.278–0.944). Differences in peak positions, slope and overall magnitude reflect structural heterogeneity in age-mixing patterns rather than differences in transmissibility.

**Figure 6.**
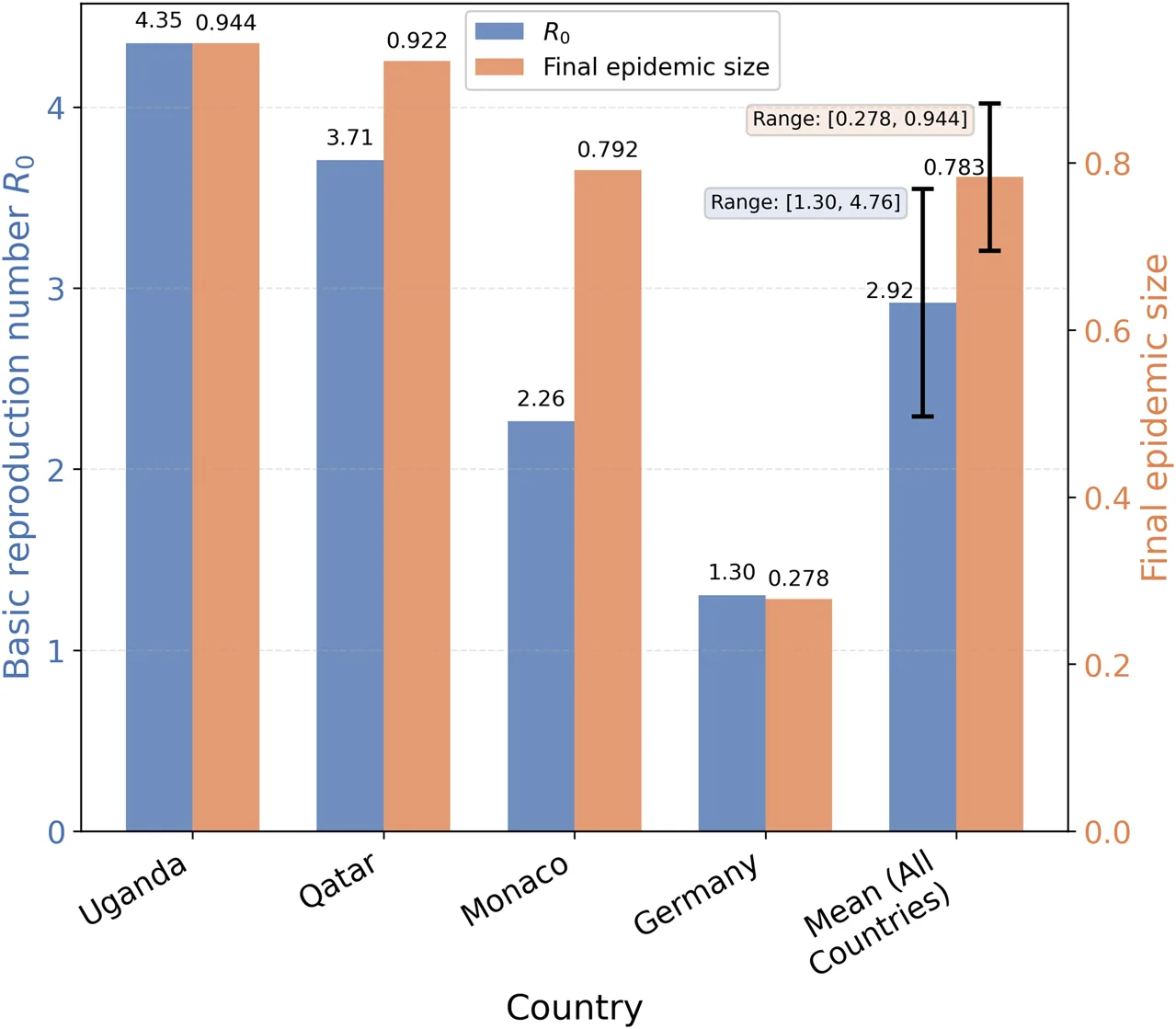
Comparison of *R*_0_ (blue bars, left axis) and final epidemic size (orange bars, right axis) for four representative countries under *β* = 0.058, *γ* = 1/3. Across all 143 countries, mean *R*_0_ = 2.918 (s.d. = 0.629, range 1.304–4.757) and mean final epidemic size = 0.783 (s.d. = 0.088, range 0.278–0.944). Error bars indicate ± 1 s.d. across all 143 countries. The four selected countries span a substantial portion of these ranges, demonstrating marked cross-country heterogeneity in both transmission potential and epidemic outcomes under identical transmission parameters.

Figure 5 presents the distribution of the final epidemic size across the 16 age groups in the four representative countries. The relationship between age and the final epidemic size is generally non-monotonic, reflecting the pivotal role of socially active age groups and cross-age mixing patterns. Uganda exhibits high infection rates across nearly all age groups, consistent with a highly connected contact structure driven by a youthful population and strong intergenerational interactions. In Qatar, the infection risk is concentrated primarily in the working-age population and declines markedly among older groups, reflecting a contact structure dominated by labour-age individuals. Germany displayed the lowest overall infection levels across all age groups, particularly among the elderly, suggesting a more age-stratified contact structure with comparatively limited intergenerational mixing. In contrast, Monaco maintained relatively high infection levels even among older age groups, indicating that infection risk among the elderly is strongly influenced by social participation and contact patterns rather than biological susceptibility alone. Collectively, these results demonstrate that the age-specific infection risk distributions across countries are fundamentally shaped by the demographic composition and structure of contact connectivity.

Figure 6 further demonstrates that the basic reproduction number *R*_0_ does not uniquely determine the final epidemic size. Countries with similar *R*_0_ values can exhibit significantly different outbreak magnitudes. This finding highlights that while *R*_0_ captures the average transmission potential, the final epidemic size is strongly influenced by heterogeneity in the age-specific contact structure. In the presence of structured mixing patterns, epidemic magnitude cannot be inferred from *R*_0_ alone.

To facilitate a systematic comparison, table 1 summarizes six key indicators for the representative countries: (i) *R*_0_, (ii) overall final epidemic size, and (iii–vi) average final infection fractions in the four aggregated age groups (young: 0–19 years, lower middle: 20–39 years, upper middle: 40–59 years and old: 60+ years). These statistics are derived from a theoretical analysis of 143 countries and regions with complete datasets. Although only the most representative countries are discussed in the main text, the full set of theoretical results for all 143 countries is provided in the Appendix for completeness and transparency. The selected countries illustrate distinct patterns: Uganda is characterized by young dominance, upper-middle dominance and the maximum final size; Qatar shows lower-middle dominance; Monaco exhibits old dominance; and Germany has the minimum final size.

**Table 1.** Main transmission characteristics of four special countries. Bold numerical values indicate the characteristic extrema among the four representative countries: the highest age-group-specific final infection fraction in each age category, and the maximum or minimum overall final epidemic size.

country	*R*_0_ value	final size	young (0–19) avg.	lower middle (20–39) avg.	upper middle (40–59) avg.	old (60+) avg.
Uganda	4.35	**0.944**	**0.976**	0.929	**0.924**	0.515
Qatar	3.71	0.922	0.905	**0.981**	0.886	0.395
Monaco	2.26	0.792	0.830	0.820	0.829	**0.723**
Germany	1.30	**0.278**	0.349	0.396	0.348	0.097

### 4.3. Stochastic simulations on age-structured contact networks

To investigate epidemic outcomes on the generated age-structured contact networks, we performed stochastic SIR simulations and compared the results with the EBCM-based analytical approximation. For each representative country, networks of size *N* = 10 000 were generated according to the procedure described in §2, and epidemic simulations were conducted with identical transmission and recovery parameters, as in the theoretical analysis.

Figure 7 compares the final epidemic sizes obtained from the stochastic network simulations with those predicted by the theoretical model. Overall, the simulation outcomes are in good agreement with the theoretical expectations, reproducing the same order of countries by epidemic magnitude. Uganda consistently exhibited the largest epidemic size, whereas Germany showed the smallest, with Qatar and Monaco occupying intermediate regimes. Minor quantitative deviations were observed, which are attributed to the finite-size effects and stochastic variability inherent to network-based simulations.

**Figure 7.**
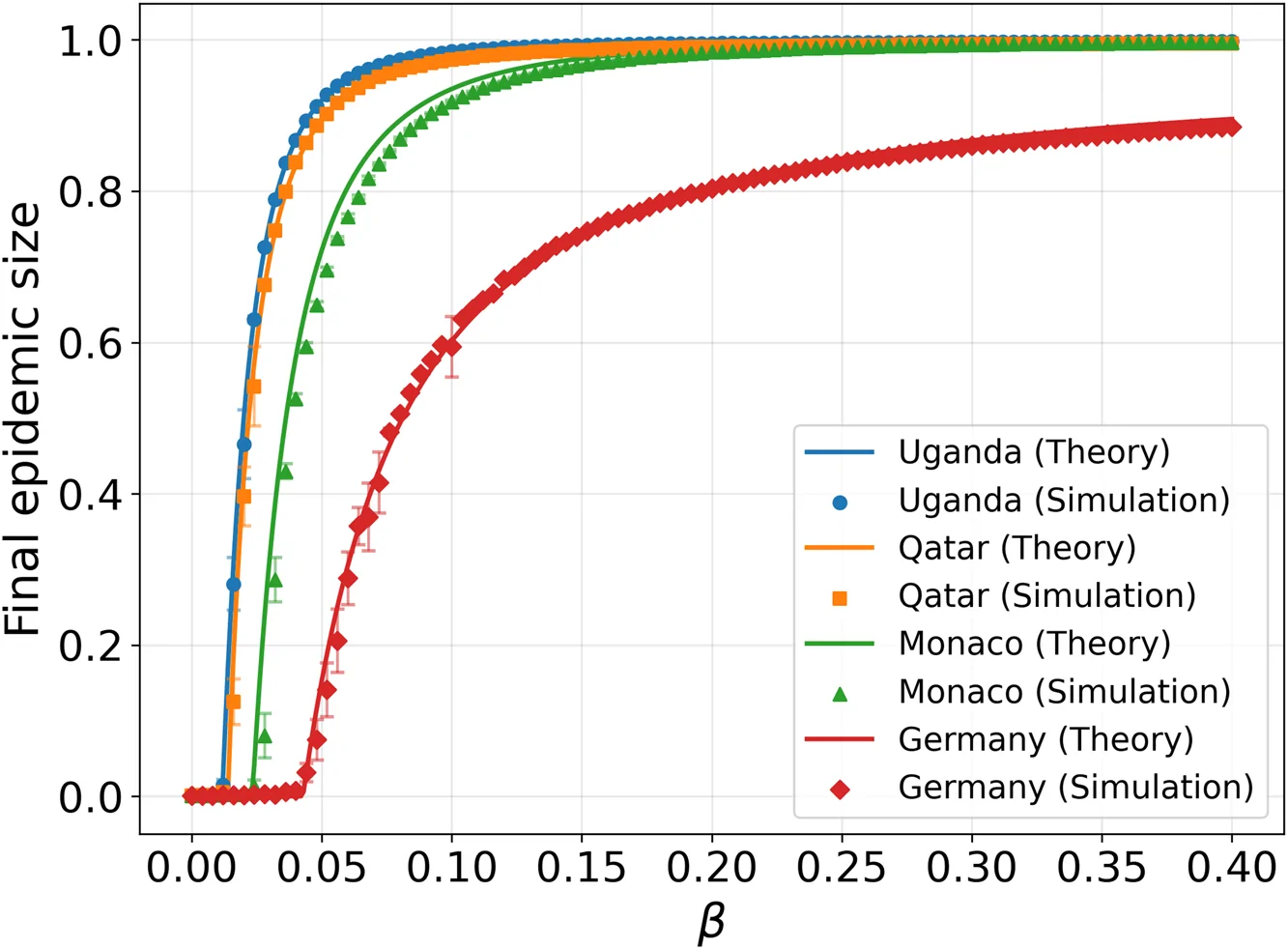
Final epidemic size obtained from stochastic SIR simulations on age-structured contact networks constructed from country-specific contact matrices. The horizontal axis represents the transmission rate *β*, and the vertical axis represents the corresponding final epidemic size. Different coloured curves correspond to different representative countries (solid lines: theoretical predictions; scatter points with error bars: simulation means from 30 independent runs). The error bars represent the 95% confidence intervals (CI), quantifying the statistical uncertainty in the estimated final epidemic size. The figure illustrates cross-country differences in epidemic responsiveness under identical recovery rate and network construction procedures.

We further investigated the sensitivity of epidemic size to the transmission rate *β*. The results indicated substantial cross-country heterogeneity in epidemic responsiveness. Uganda exhibits rapid epidemic growth even at relatively low values of *β*, whereas Germany maintains comparatively small epidemics across a broader parameter range. These differences reflect variations in demographic structure and contact intensity, which shape the effective connectivity of the underlying networks.

To better understand age-dependent transmission patterns, we analysed the final epidemic sizes stratified into four aggregated age categories: younger (0–19 years), lower middle-aged (20–39 years), upper middle-aged (40–59 years) and older (60+), aggregated from the original 16 five-year age groups. The age-stratified simulation results are shown in figure 8. The simulation results are consistent with the patterns identified in the theoretical analysis. Specifically, Uganda displayed the highest infection burden in younger and upper-middle-aged groups, Qatar showed a pronounced concentration of infections in the lower-middle-aged group, and Monaco exhibited the highest relative infection burden among older individuals. Germany has consistently lower infection rates across all age groups.

**Figure 8.**
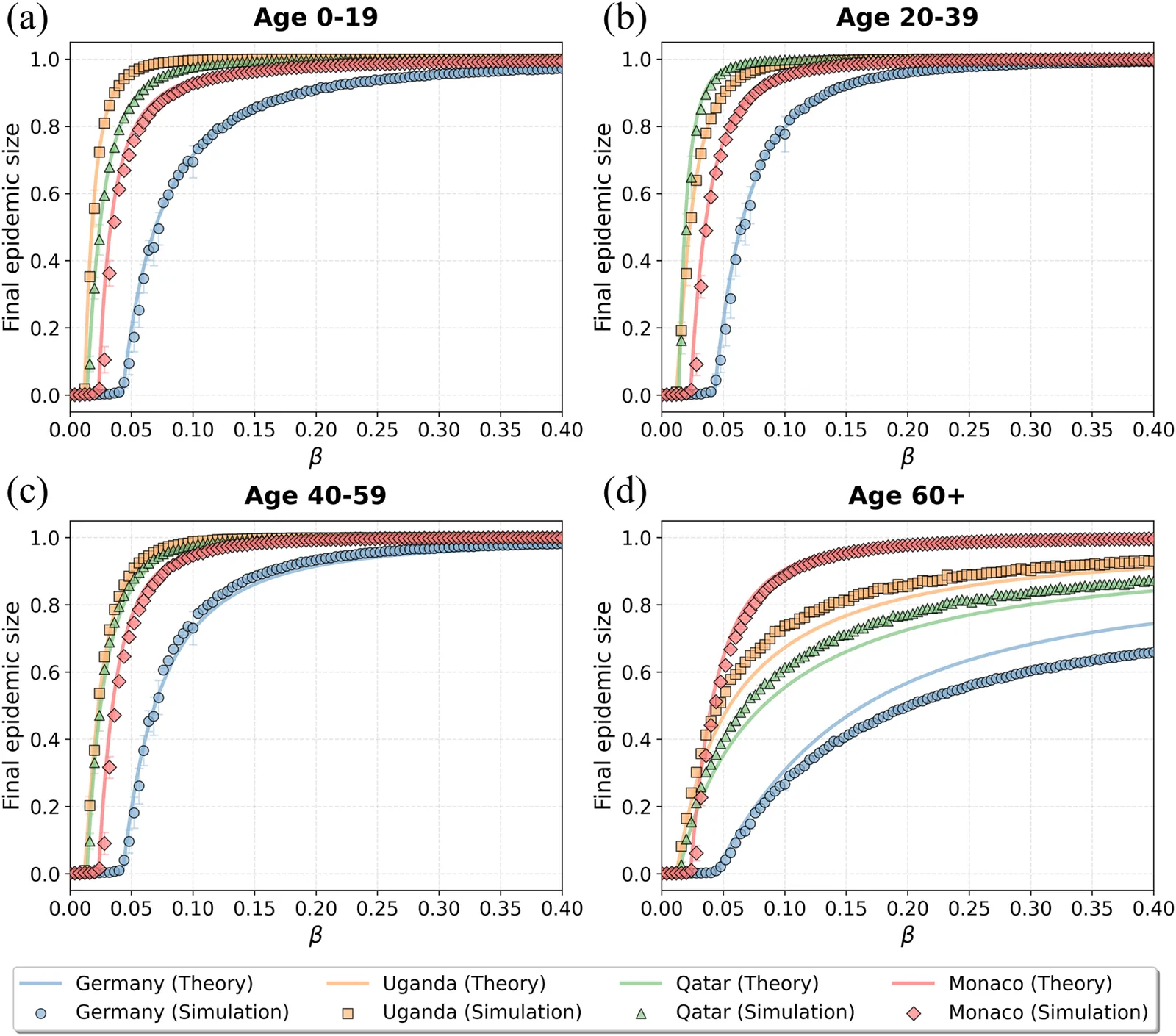
Age-stratified final epidemic size obtained from stochastic SIR simulations on country-specific age-structured contact networks. Panels (a)–(d) correspond to four aggregated age categories: (a) 0–19 years, (b) 20–39 years, (c) 40–59 years, and (d) 60+ years. In each panel, the horizontal axis represents the transmission rate *β*, and the vertical axis represents the final epidemic size within the corresponding age category. Different coloured curves denote different representative countries (solid lines: theoretical predictions; scatter points with error bars: simulation means with 95% CI). The figure highlights how age-dependent contact structures lead to distinct transmission sensitivities across demographic groups.

These results confirmed that the stochastic network simulations preserved the qualitative transmission patterns predicted by the analytical framework. They demonstrated that differences in age composition and contact structure alone, under identical epidemiological parameters, were sufficient to generate markedly distinct epidemic regimes. The comparison indicates that the EBCM-based approximation captures the qualitative patterns observed in the stochastic network simulations.

### 4.4. Impact of school or workplace closures on epidemic transmission

To quantify the structural contributions of different social settings to epidemic transmission, we examined intervention scenarios in which specific contact layers were removed from the full age-specific contact matrix. The baseline contact matrix for each country was defined as $\tilde{C}=C^{home}\;+C^{school}+C^{work}+C^{other}$, where each component represents the setting-specific contact matrix provided by Prem *et al.* [36]. Two intervention scenarios were considered: school-contact removal: $\tilde{C}-C^{school}$ and workplace-contact removal: $=\tilde{C}\;-C^{work}$. For each scenario, both the theoretical age-structured model and the stochastic network-based simulations were recomputed using the modified contact matrix, while keeping the transmission rate *β* and recovery rate *γ* fixed at their calibrated values. This approach isolates the structural effect of removing specific interaction layers without altering the pathogen transmissibility.

To understand how school and workplace closures affect epidemic transmission, we analyse the structural changes they induce in the underlying age-structured contact networks. Figure 9 quantifies four key metrics—cross-age edge ratio, average shortest path length, mean degree and largest component fraction—under baseline, school-removal and workplace-removal scenarios for the four representative countries.

**Figure 9.**
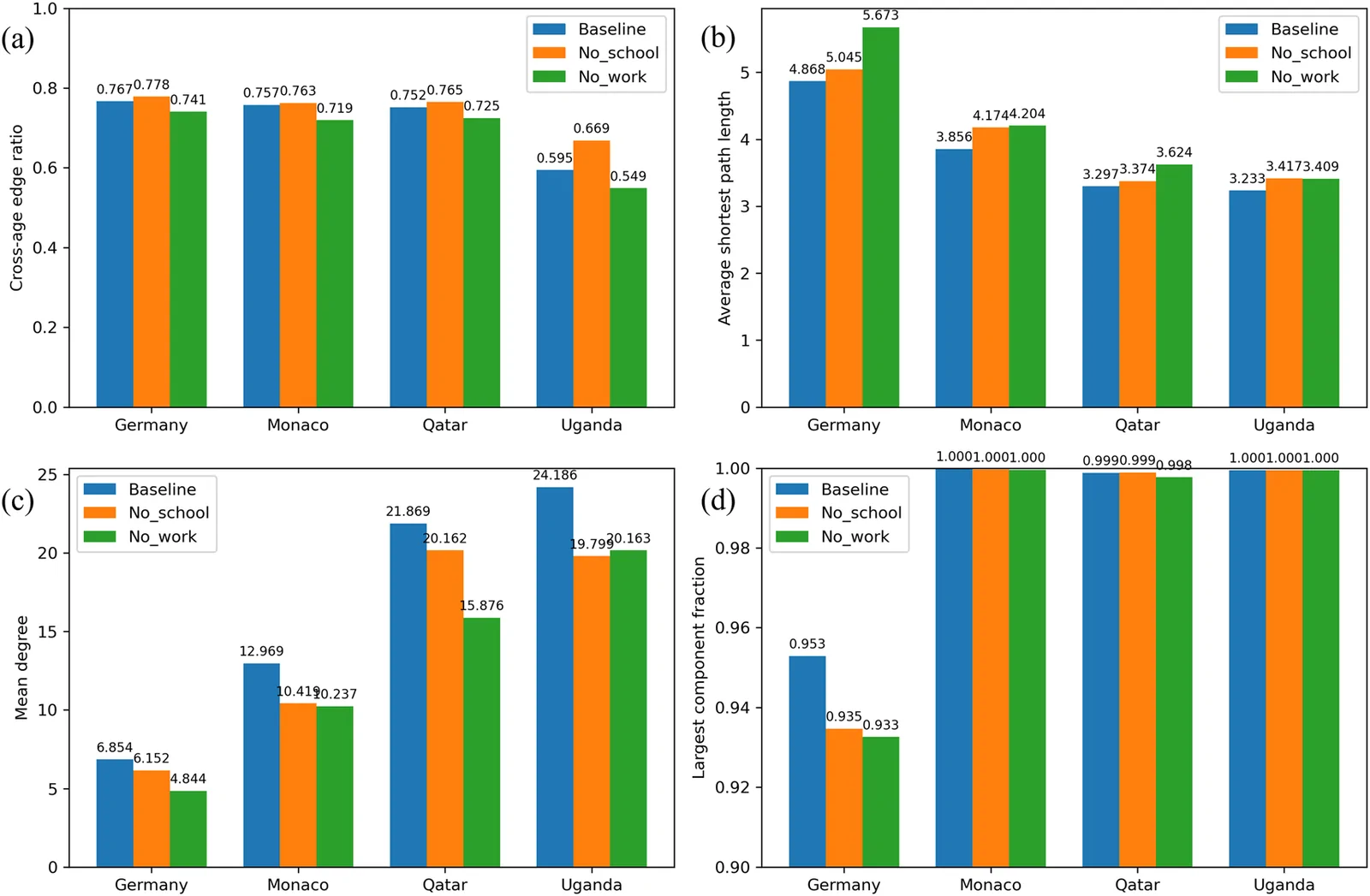
Structural changes in age-structured contact networks under school- and workplace-closure scenarios for four representative countries (Germany, Monaco, Qatar and Uganda). For each country, three intervention scenarios are compared: baseline (blue), school removal (orange) and workplace removal (green). Panels show (a) cross-age edge ratio (proportion of edges connecting different age groups), (b) average shortest path length (computed on the largest connected component), (c) mean degree and (d) fraction of nodes in the largest connected component. Workplace removal consistently reduces cross-age connectivity and mean degree, increases path length and slightly fragments the network in Germany, whereas school removal has weaker and country-dependent effects.

Workplace closure consistently reduces the cross-age edge ratio across all countries (Germany: −3.4%; Monaco: −5.0%; Qatar: −3.6%; Uganda: −7.7%), indicating that workplace contacts act as essential bridges between age groups. This is accompanied by a marked decrease in mean degree (e.g. Germany: −29.4%) and an increase in average shortest path length (e.g. Germany: +16.4%), reflecting network sparsification and longer transmission chains. The largest component fraction remains above 0.93 in all scenarios, but Germany shows a slight reduction under workplace closure (from 0.953 to 0.933), indicating mild fragmentation. These structural changes explain why workplace closure achieves stronger epidemic suppression (figures 10–12): it directly dismantles intergenerational bridges and lengthens transmission pathways.

**Figure 10.**
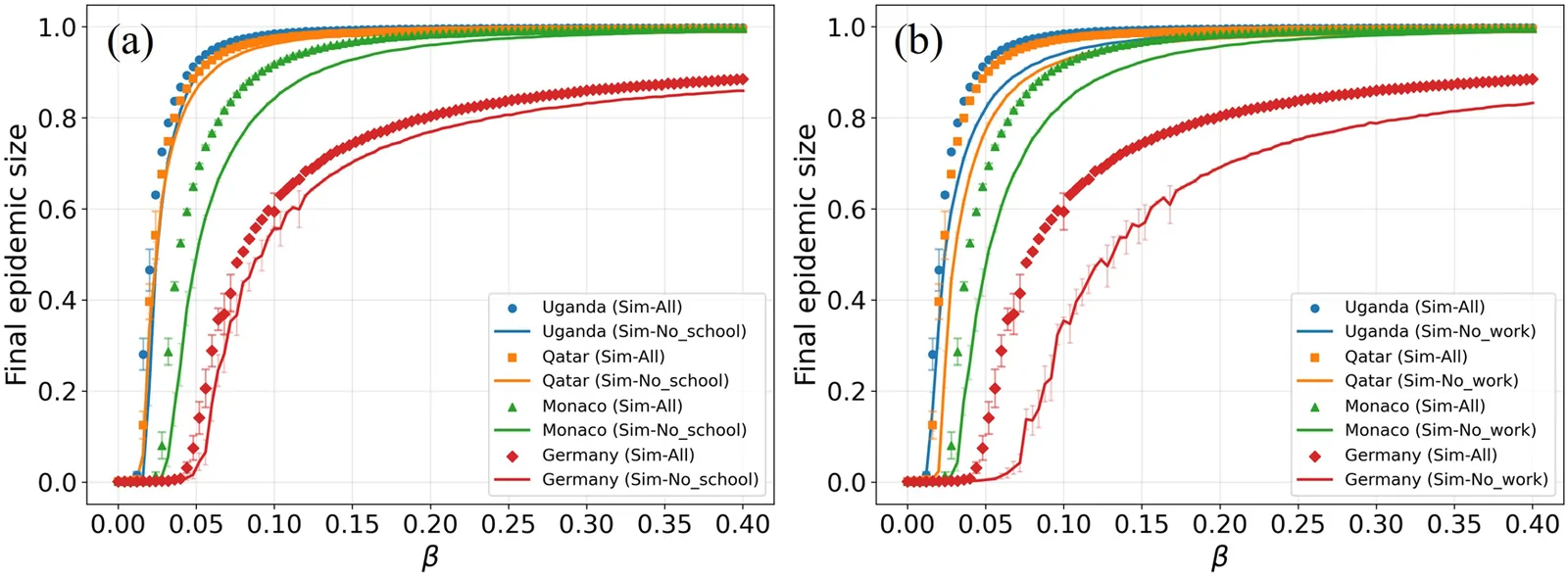
Final epidemic size as a function of the transmission rate *β* under setting-specific contact-removal scenarios for four representative countries. (a) Final epidemic size versus *β* after removing school-related contacts from the full age-specific contact matrix. (b) Final epidemic size versus *β* after removing workplace-related contacts. The recovery rate is fixed at *γ* = 1/3. Solid lines with error bars represent the mean and 95% confidence interval (CI) for the intervention scenarios; scatter points with error bars represent the mean and 95% CI for the baseline scenario (all contacts). Different colours correspond to different countries. Error bars are derived from 30 independent stochastic runs. Removing workplace contacts produces a substantially larger reduction in the final epidemic size than removing school contacts, particularly at intermediate transmission rates and in countries where older adults dominate contact structures (e.g. Germany and Monaco). The relative importance of intervention strategies thus strongly depends on the underlying age structure and setting-specific mixing patterns.

**Figure 11.**
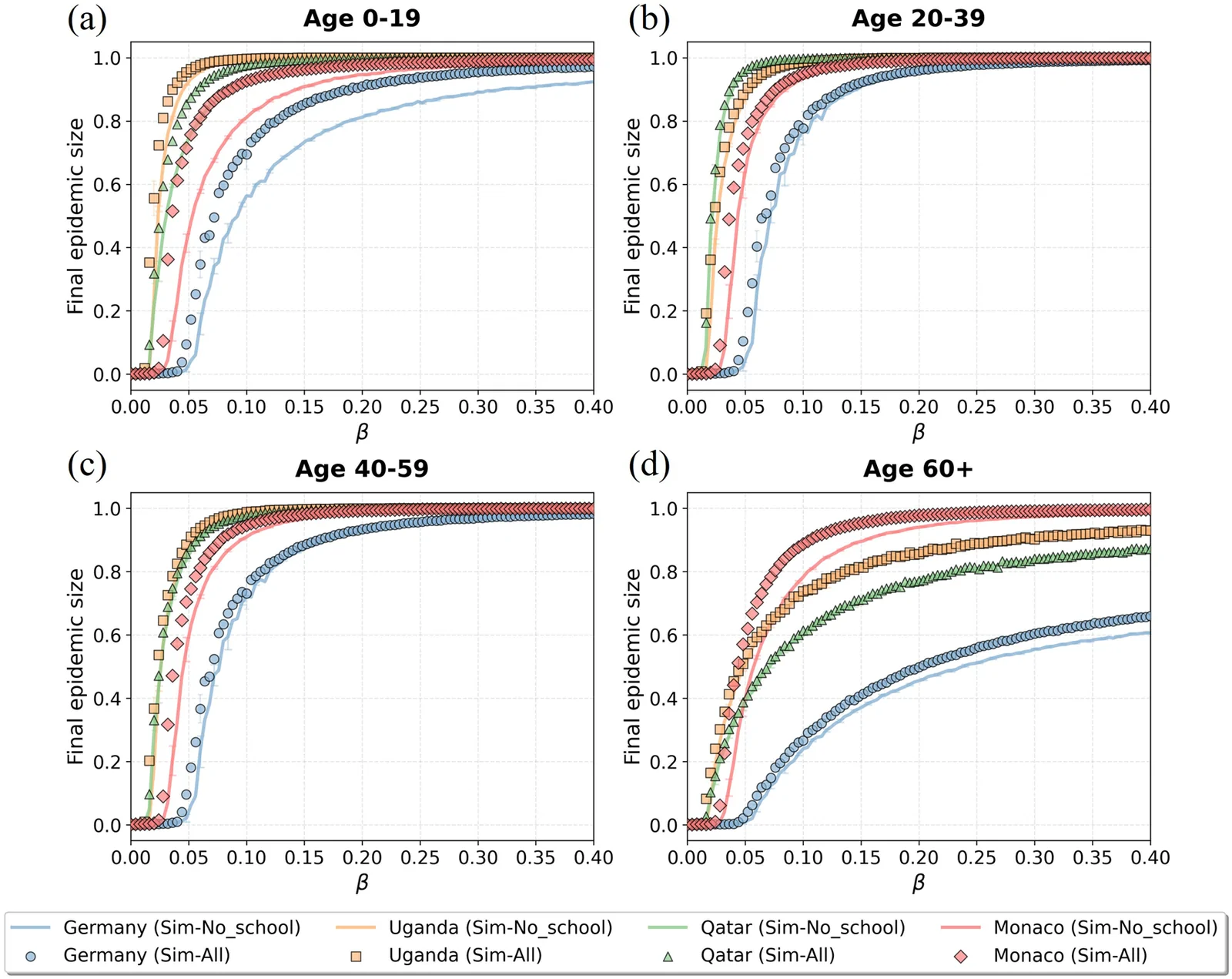
Age-stratified final epidemic size under the school-closure scenario (school contacts removed) for the four representative countries. Panels (a)–(d) correspond to four aggregated age categories: (a) 0–19 years, (b) 20–39 years, (c) 40–59 years, and (d) 60+ years. Each panel shows the final infection fraction across age groups as a function of the transmission rate *β*, with the recovery rate fixed at *γ* = 1/3. Solid lines with error bars represent the mean and 95% confidence interval (CI) for the intervention scenarios; scatter points with error bars represent the mean and 95% CI for the baseline scenario (all contacts). The 0–19 age group is the most affected by the removal of school-related contacts. Differences across countries reflect variations in demographic structure and age-specific mixing patterns.

**Figure 12.**
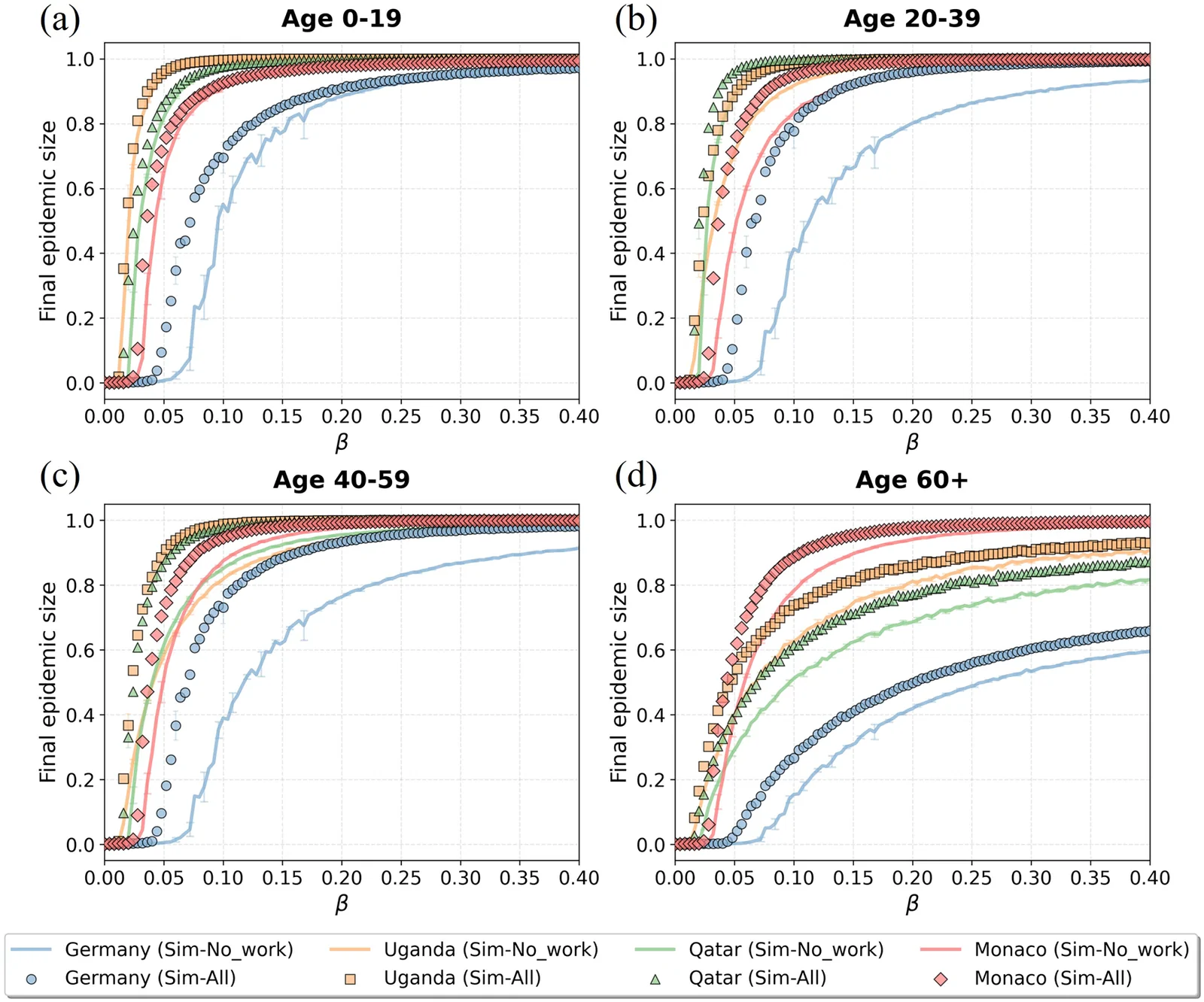
Age-stratified final epidemic size under the workplace-closure scenario (workplace contacts removed) for the four representative countries. Panels (a)–(d) correspond to four aggregated age categories: (a) 0–19 years, (b) 20–39 years, (c) 40–59 years, and (d) 60+ years. Each panel shows the final infection fraction across age groups as a function of the transmission rate *β*, with the recovery rate fixed at *γ* = 1/3. Solid lines with error bars represent the mean and 95% confidence interval (CI) for the intervention scenarios; scatter points with error bars represent the mean and 95% CI for the baseline scenario (all contacts). The 20–59 age groups are the most affected by the removal of workplace-related contacts. Differences across countries reflect variations in demographic structure and age-specific mixing patterns.

School closure produces weaker and more heterogeneous effects. In Germany, Monaco and Qatar, the cross-age edge ratio increases marginally (+1.5%, +0.7% and +1.8%, respectively), while in Uganda it rises more substantially (+12.5%). This increase does not mean that the absolute number of cross-age contacts becomes larger. Instead, it arises because school contacts are predominantly age-assortative (i.e. between individuals of the same age group). Removing these edges drastically reduces the total number of edges while leaving most cross-age edges (from home, community and workplace) intact. As a result, the proportion of cross-age edges increases even though their absolute count may decline slightly. For example, in Uganda the absolute number of cross-age edges decreases from approximately 71 900 to 66 200 (cross edge ratio multiplied by the number of edges) under school closure, but the total number of edges drops even more sharply (from 120 929 to 98 995), causing the ratio to rise. This effect is most pronounced in Uganda because its school contacts are highly age-concentrated and its baseline cross-age ratio is low. The modest or negligible reductions in mean degree and path length under school closure further indicate that school removal does not substantially disrupt inter-age transmission bridges. Consequently, school closure alone has a limited impact on overall epidemic size, as seen in figures 10–12.

These structural analyses demonstrate that network-based simulations uncover intervention mechanisms—such as the selective removal of age-assortative versus bridging edges—that remain hidden in mean-field models relying solely on average contact rates.

Figure 10 presents the final epidemic size as a function of the transmission rate *β* under the baseline and intervention scenarios. Removing workplace contacts produced a substantially larger reduction in the final epidemic size than removing school contacts, particularly at intermediate transmission rates. This effect is most pronounced in countries where older adults dominate contact structures, such as Germany and Monaco. By contrast, school-contact removal yielded a comparatively modest reduction in the overall epidemic size. Although school contacts contribute to transmission among younger populations, their removal alone does not substantially alter the global connectivity of contact networks in most countries. These results indicate that the relative importance of intervention strategies strongly depends on the underlying age structure and setting-specific mixing patterns.

To better understand the structural mechanisms underlying these differences, we examined the age-specific final epidemic sizes under each intervention scenario (figures 11 and 12). Under the school-contact removal scenario, reductions in the infection burden were primarily concentrated in younger age groups (0–19 years). The impact on middle-aged and older groups remained limited, and the overall reduction in epidemic size was moderate. This reflects that school contacts are largely assortative within younger cohorts and contribute less to cross-generational transmission. In contrast, under the workplace-contact removal scenario, a pronounced reduction in the infection burden was observed in the 20–59 age range. Importantly, this reduction was indirectly propagated to older age groups through decreased cross-age transmission. Therefore, the overall epidemic size was substantially suppressed compared with the school-removal scenario. These findings suggest that workplace contacts play a more structurally central role in sustaining transmission chains across multiple age groups, whereas school contacts primarily amplify transmission in younger cohorts.

The differential impacts of these interventions can be understood in terms of network topology. Workplace interactions typically generate dense connectivity within working-age populations and serve as bridges between age groups. Therefore, removing this layer reduced both intragroup clustering and intergroup connectivity. In contrast, school contacts are strongly age-assortative and largely confined to younger classes. Their removal decreases the local transmission intensity but has a weaker effect on global network connectivity. Taken together, these results demonstrate that the epidemiological consequences of non-pharmaceutical interventions critically depend on how different social layers contribute to age-structured contact networks. Therefore, evaluating intervention effectiveness requires explicit consideration of demographic composition and setting-specific mixing patterns rather than relying solely on population-averaged transmission parameters.

## 5. Conclusion and discussion

This study investigated how age-specific contact structures shape epidemic dynamics by integrating data-driven contact matrices with both mean-field and network-based epidemic models. By applying a unified transmission rate across countries, we demonstrated that substantial cross-country heterogeneity in epidemic outcomes can emerge solely from differences in demographic composition and contact topology.

Our results show that the basic reproduction number alone does not uniquely determine the impact of the epidemic. Countries with comparable values of *R*_0_ may exhibit markedly different final epidemic sizes and age-specific infection burdens. This discrepancy arises from the structural heterogeneity embedded in age-dependent contact patterns and the resulting degree distributions of the constructed networks. Variations in intergenerational mixing and within-group contact intensity play decisive roles in shaping transmission pathways and in amplifying or constraining the magnitude of outbreaks.

The comparative analysis of representative countries highlights that epidemic risk profiles are strongly modulated by demographic structures. Younger populations with dense cross-age contact tend to sustain broader transmission across age groups, whereas ageing populations, characterized by more stratified contact patterns, exhibit a comparatively constrained epidemic spread. These differences persist even under identical transmission parameters, underscoring the importance of explicitly incorporating contact heterogeneity in epidemic modelling.

The analysis of school- and workplace-contact removal further illustrates that intervention effectiveness critically depends on setting-specific contact structures. School-based interventions primarily affect transmission within younger age groups, whereas workplace-related contacts contribute more substantially to cross-age transmission and the overall epidemic magnitude. These findings suggest that non-pharmaceutical interventions should be evaluated in the context of country-specific demographic and contact configurations rather than assuming uniform effects.

This study had several limitations. First, the contact matrices represent population-averaged daily contact and do not capture temporal variations or behavioural adaptations. Second, the per-contact transmission rate was assumed to be identical across countries, isolating structural effects but neglecting biological and contextual variability. Third, network simulations were conducted on finite-size synthetic networks that could introduce stochastic variability. Despite these limitations, this framework provides a systematic approach for quantifying potential epidemic risks based solely on demographic and contact structure data.

Future work may extend this framework by incorporating behavioural adaptation, temporal contact variation or empirical epidemic data for calibration and validation. Such extensions would further enhance the predictive capacity of age-structured network models and contribute to more context-sensitive public health decision-making.

## Supplementary Material



## Data Availability

All data and code supporting this study are publicly available at Zenodo: https://zenodo.org/records/21486658. This repository contains the theoretical calculation data, simulation results and figure-generation scripts used in the present manuscript. Supplementary material is available online [61].
