# Supplementary material for: Beyond mean-field age-structured models: degree heterogeneity and cross-country variability in epidemic risk
Source: J R Soc Interface. 2026 Oct 21;23(243):20260225. doi: 10.1098/rsif.2026.0225 (PMC13682900; doi:10.1098/rsif.2026.0225)
Supplement: Supplementary file 1 [file rsif.2026.0225.zip › rsif.2026.0225/rsif20260225_si_001.docx]

**Figure S1.** Age-stratified final epidemic size obtained from stochastic SIR simulations on country-specific age-structured contact networks. The 16 panels correspond to 16 age groups (Age 1 to Age 16, each representing a 5-year interval from 0–4 years to 75+ years). In each panel, the horizontal axis represents the transmission rate β, and the vertical axis represents the final epidemic size within the corresponding age group. Solid lines represent theoretical predictions from the edge-based mean-field model, while scatter points with error bars represent simulation means with 95% confidence intervals (CI) from 30 independent stochastic runs. Different colours denote different representative countries. The figure illustrates how age-dependent contact structures lead to distinct transmission sensitivities across fine-grained age groups.

**Figure S2.** Age-stratified final epidemic size under two simulation scenarios (baseline: all contacts; intervention: school closure) for 16 age groups. The 16 panels correspond to 16 age groups (Age 1 to Age 16, each representing a 5-year interval from 0–4 years to 75+ years). In each panel, the horizontal axis represents the transmission rate β, and the vertical axis represents the final epidemic size within the corresponding age group. Solid lines with error bars represent the school-closure scenario (mean ± 95% CI), while scatter points with error bars represent the baseline scenario (all contacts) with 95% confidence intervals from 30 independent stochastic runs. Different colours denote different representative countries. The figure illustrates how removing school contacts disproportionately reduces infection burden in school-age groups, while older age groups remain largely unaffected, reflecting age-dependent contact structures and transmission pathways.

**Figure S3.** Age-stratified final epidemic size under two simulation scenarios (baseline: all contacts; intervention: workplace closure) for 16 age groups. The 16 panels correspond to 16 age groups (Age 1 to Age 16, each representing a 5-year interval from 0–4 years to 75+ years). In each panel, the horizontal axis represents the transmission rate β, and the vertical axis represents the final epidemic size within the corresponding age group. Solid lines with error bars represent the school-closure scenario (mean ± 95% CI), while scatter points with error bars represent the baseline scenario (all contacts) with 95% confidence intervals from 30 independent stochastic runs. Different colours denote different representative countries. The figure illustrates how removing workplace contacts disproportionately reduces infection burden in working-age adults (20–59 years), while younger and older age groups are less directly affected, reflecting adult-dominated contact structures and transmission pathways under workplace closures.
